# Uterine Fibroids and Progestogen Treatment: Lack of Evidence of Its Efficacy: A Review

**DOI:** 10.3390/jcm9123948

**Published:** 2020-12-05

**Authors:** Jacques Donnez

**Affiliations:** 1 Société de Recherche pour l’Infertilité (SRI), 1150 Brussels, Belgium; jacques.donnez@gmail.com; 2 Université Catholique de Louvain, 1200 Brussels, Belgium

**Keywords:** uterine fibroids, progesterone, progestogen, GnRH agonist, GnRH antagonist, heavy menstrual bleeding, add-back therapy

## Abstract

Background: The objective of this review is to determine the evidence or, conversely, the absence of evidence regarding the effectiveness of progestogens in treating premenopausal women with uterine fibroids. In particular, the goal is to address recurring questions as to whether they are effective or not for managing symptoms commonly attributed to fibroids. Methods: A review of the most relevant papers (*n* = 63) on the efficacy of progesterone and progestogens as medical therapy for uterine fibroids. Results: Having reviewed the most significant papers on the relationship between uterine fibroids and progesterone/progestogens, it is clear that there is biochemical, histological and clinical evidence that progesterone and progestogens play a critical role in the pathogenesis of myomas. Conclusion: Since progesterone is already implicated in the pathogenesis of this entity, using progestogens to manage fibroids is like constantly adding fuel to the fire, rendering this treatment ineffective.

## 1. Introduction

The prevalence of fibroids depends upon ethnic background [1,2]. It varies widely based on the diagnostic approach, but is estimated to be more than 60% in women over the age of 45 years [3,4,5].

While some fibroids are asymptomatic, others result in symptoms that warrant therapy [3,6]. The most common symptom is heavy menstrual bleeding (HMB), but pelvic pain, bulk symptoms and infertility are other frequent manifestations that may greatly affect the quality of life of these women [2,4,6,7].

### 1.1. HMB: The Most Common Complaint

A number of theories have been proposed to explain fibroid-related HMB. These include an increase in uterine surface area, endometrial ulceration or an enlarged vascular network on the surface of a submucosal fibroid, greater vascular flow into the myometrium, changes in contractility of the inner junctional zone, and congestion of the endometrium and myometrium by compression of the myometrial venous plexus [3]. Dysregulation of normal myometrial vascular function in uterine fibroids and surrounding myometrium is due to anomalies in the expression of angiogenic growth factors and their receptors [8,9]. The presence of uterine fibroids may also impact the composition of the overlying endometrium, particularly the number of uterine natural killer cells and macrophages [10,11], which are potential producers of angiogenic growth factors.

As stressed by Ikhena and Bulun, uterine fibroids significantly affect gene expression in the endometrium [12]. The consecutive roles of transforming growth factor beta-3 (TGF-β3) and HOXA-10, leading to impaired endometrial receptivity, were first suggested by Rackow and Taylor [13]. TGF-β is known to be elevated in leiomyomas and acts as a diffusible signaling molecule to alter bone morphogenetic protein 2 (BMP-2), reducing HOXA-10 expression throughout the endometrium [14,15]. Moreover, Sinclair et al. and Taylor both identified defective decidualization and hemostasis in the endometrium, which may partially explain the heavier bleeding in women with uterine fibroids [15,16].

Despite significant advances in understanding the molecular changes in leiomyomas and associated myometrium and endometrium, it remains unclear why clinical symptoms are so diverse. Considering the strong association between uterine fibroids and HMB, ultrasound should be performed as a wholly appropriate diagnostic approach.

### 1.2. Existing Therapeutic Approach

Numerous treatments are available, including pharmacological, surgical, and radiological interventions, such as uterine embolization [17] or MRI-guided focused ultrasound [2]. However, as reported in a recent editorial, hysterectomy still remains the go-to one-size-fits-all treatment for uterine fibroids. Nonetheless, many women need an effective alternative to hysterectomy for various reasons, including faster recovery and maintenance of fertility [18]. There is therefore a need for conservative options, and safe and effective medical therapy is one of them.

Among existing medical therapies, tranexamic acid, combined oral contraceptives, oral and injectable progestogens, progestogen-releasing intra uterine systems, antiprogesterone, gonadotropin-releasing hormone (GnRH) agonists and antagonists, selective progesterone receptor modulators (SPRMs), selective estrogen receptor modulators (SERMs), aromatase inhibitors, danazol and gestrinone are frequently cited [2,3,6,7].

The majority of these therapies are used for the management of abnormal uterine bleeding but are not specifically indicated for uterine fibroids. Among them, the most commonly used are progestogens.

### 1.3. The Recurring Question: Are Progestogens Effective?

A recent decision by the EMA’s human medicines committee (CHMP) recommended restricting the use of medicines containing 5 mg ulipristal acetate as they were linked to cases of serious liver injury. If confirmed by the European Commission, this may push gynecologists to go on prescribing progestogens to treat uterine fibroids, so it is high time to evaluate their efficacy.

Progestogen is a natural or synthetic hormone. Progesterone is a natural hormone secreted by the corpus luteum, while progestin is a synthetic progestogen that can be administered orally, vaginally or by intramuscular injection. Progestogens have been used all over the world for many years in the management of uterine fibroids, despite the lack of evidence and absence of adequately designed and powered studies. One of them, depot medroxyprogesterone acetate (DMPA), has been approved for use in more than 100 countries, but other progestogens (lynestrenol, pregnane and nor-pregnane) are still used in uterine fibroids therapy.

The objective of this review is to determine the evidence or otherwise regarding the effectiveness of progestogens in treating premenopausal women with uterine fibroids. In particular, we will try to address recurring questions as to whether they are effective or not for managing symptoms commonly attributed to fibroids.

**Figure 1:**
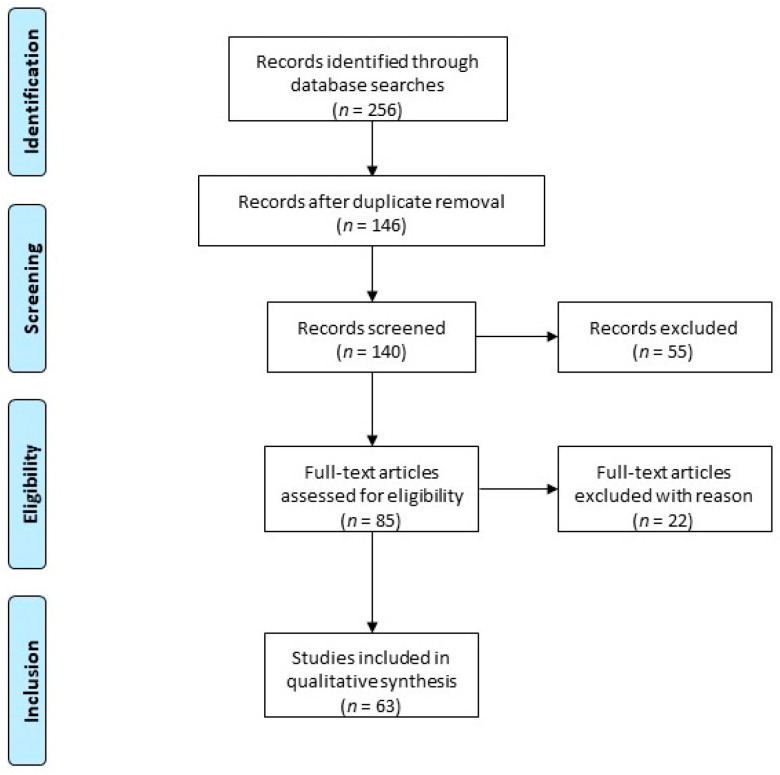
Article disposition flow diagram: The following keywords were used: uterine fibroids, progesterone, progestogen, GnRH agonist, GnRH antagonist, heavy menstrual bleeding, add-back therapy. Two hundred and fifty-six records were identified. After duplicate removal, 140 records were screened, of which only peer-reviewed articles focusing on the subject were considered for eligibility assessment (*n* = 63). Among the conducted studies, specific various criteria led to the exclusion of 22 papers due to duplicated results.

A literature search was conducted through an electronic database (PubMed, Embase, the Cochrane library) up to September 2020. The following key words were entered: uterine fibroids, progesterone, progestogen, GnRH agonist, GnRH antagonist, heavy menstrual bleeding, add-back therapy (Figure 1).

## 2. Biochemical and Histological Evidence Supporting the Critical Role of Progesterone and Progestogens in the Pathogenesis of Myomas

Traditionally, estrogen has been considered the major promoter of myoma growth, but the role of progesterone has become increasingly obvious over the years. Back in 1949, elevated mitotic activity was observed in uterine fibroids removed from women treated with 20 mg of progesterone daily for 1 to 6 months [19]. In the 1980s, higher mitotic activity was confirmed in myomas treated with medroxyprogesterone acetate (MPA) [20] and in those in the secretory phase compared to the proliferative phase [21].

During the early 1990s, Lamminen et al. showed that the proliferation index in fibroids from postmenopausal women receiving estrogen and progestin was higher than that in myomas removed from postmenopausal women given estrogen alone [22]. By the late 1990s, the crucial role of progesterone was abundantly clear. A number of studies reported greater expression of both progesterone receptor A (PR-A) and progesterone receptor B (PR-B) in leiomyoma tissue [23,24] than in adjacent normal myometrium. Moreover, higher proliferative activity, evidenced by proliferating cell nuclear antigen (PCNA) and the mitotic index, was encountered in leiomyomas during the luteal (secretory) phase [24] compared to the proliferative phase.

During the last decade, Kim et al. proved that progesterone promotes growth of uterine fibroids by increasing proliferation, cellular hypertrophy and deposition of the extracellular matrix (ECM) [25]. In an extensive review, Moravek et al. concluded that progesterone and progestin play key roles in uterine fibroid growth [26]. Ishikawa et al. determined that estrogen alone is not an in vivo mitogen, but plays a permissive role, acting via the induction of PR expression and thereby allowing leiomyoma responsiveness to progesterone [27,28]. Concentrations of PR-A and PR-B proteins were also found to be higher in leiomyomas than in matched myometrium [29].

**Figure 2:**
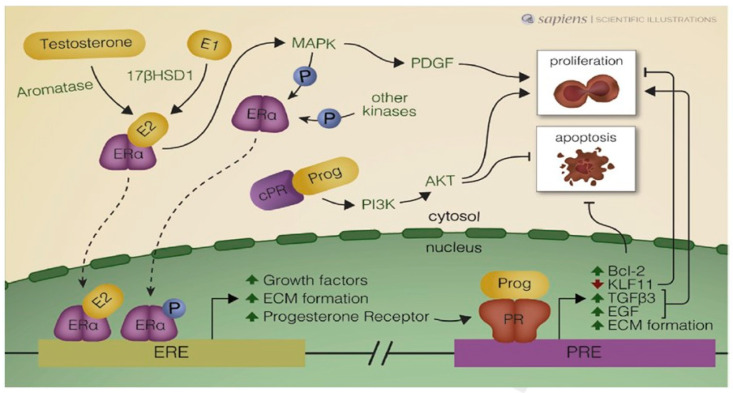
Schematic illustration of autocrine and paracrine mechanisms activated by estrogen receptor alpha (Era) and progesterone receptors (PRs) in uterine leiomyoma cells. Estradiol (E2) arrives with the blood supply (endocrine), but is also synthesized within cells (autocrine), from precursors such as testosterone and estrone (E1). ERa may be phosphorylated (P) by kinases and interact with estrogen response elements (EREs) in the nucleus. 178HSD1: 178-hydroxysteroid dehydrogenase type 1; MAPK: mitogen-activated protein kinase: PDGF: platelet-derived growth factor; P13K: phosphatidylinositol-3-kinase; AKT: serine/threonine protein kinase: Bcl-2: B-cell leukemia/lymphoma-2 protein; KLF: Kruppel-like transcription factor 11; TGF-83: transforming growth factor beta 3;EGP: epidermal growth factor; ECM: extracellular matrix; Prog: progesterone; R: progesterone receptor in the cytosol and PRE: progesterone response element. *From Reis* et al. *Best Practice & Research, Clinical Obstetrics and Gynaecology (2015), with permission from the editor and author.*

Kim and Sefton and Reis et al. described activation of signaling pathways in uterine fibroids by both estrogen and progesterone [30,31]. Progesterone is able to cause rapid membrane-initiated effects, independent of gene transcription, which alter the production of second messengers involved in cell signaling transduction pathways. The PI3K/AKT pathway is mediated by progesterone, which can quicky activate this pathway through its receptors. PTEN, on the other hand, should be considered a negative regulator of AKT [30]. Progesterone and growth factor signaling pathways are interconnected and govern numerous physiological processes, such as proliferation, apoptosis and differentiation (Figure 2).

As illustrated in Figure 2, numerous autocrine and paracrine mechanisms are activated by ERα and PRs in leiomyoma cells, demonstrating the crucial role of progesterone and progestogens in the pathogenesis of uterine fibroids.

## 3. Clinical Evidence Supporting the Critical Role of Progesterone and Progestogens in the Pathogenesis of Myomas

### 3.1. Progestogens as Medical Therapy for Myomas

Back in the early 1960s, it was reported that 15 out of 16 patients with uterine myomas treated with a synthetic progestin (norethynodrel, 20 to 40 mg daily) showed significantly enlarged uterine myomas, which returned to pretreatment size after discontinuation of progestin therapy in 70% of cases [32]. Only one randomized clinical trial (RCT), comparing lynestrenol and GnRHagonist, was published by Verspyck et al. (2000). This study showed that there was a statistically significant reduction in mean uterine fibroid volume at 16 weeks in the leuprolin group (26.5 +/− 4.5%) compared to lynestrenol (7.3% +/− 5%). However, as pointed out by Sangkomkamhang et al., the quality of the study was very low. Indeed, the risk of bias was judged to be high due to many patients being lost to follow-up (up to 22.7% in the lynestrenol group vs. 9.3% in the leuprolin group) [33].

Other studies were performed in France with promegestrone (Surgestone^®^) [34] and nomegestrol acetate [35]. Neither was able to demonstrate any significant reduction in bleeding or myoma size in most cases. Indeed, no individual study has established that progestogens have a beneficial effect on the different pathogenetic mechanisms involved in fibroid related HMB.

Moreover, Boyd and McCluggage described morphological changes induced by progestogens in myomas. They included small and/or large areas of infarct-type necrosis, with increased surrounding cellularity, mitotic activity, nuclear pyknosis, cytoplasmic eosinophilia, epithelioid morphology, stromal edema, hemorrhage myxoid changes and inflammatory infiltrates, including granulated lymphocytes. As stressed by the authors, pathologists should be aware of these progestogen-associated changes, since erroneous diagnoses of leiomyosarcoma or smooth muscle tumor of uncertain malignant potential cell (STUMP) may otherwise be reached [36].

### 3.2. Association of GnRH Agonist and Add-Back Therapy

Several clinical trials evaluating the association of GnRH agonist plus add-back therapy strongly suggested an important role for progesterone and progestogens in myoma growth. Friedman et al. demonstrated that there were no significant changes in myoma volume during cotreatment with GnRH agonist plus MPA, although a significant reduction was observed in patients treated with GnRH agonist alone (leuprolide) [37]. These authors concluded that MPA appears to inhibit the ability of GnRH agonist to shrink uterine myomas. In an RCT, Friedman et al. showed that high doses of norethindrone can reverse the effectiveness of GnRH agonist induced myoma shrinkage in a dose-dependent manner [38].

In another RCT, Carr et al. compared the effectiveness of administering MPA (20 mg/day) along with GnRH agonist (leuprolide acetate 1 mg/day subcutaneously) [39]. Total uterine volume, as determined by magnetic resonance imaging, decreased to 73% of the baseline at 12 weeks (*p* < 0.04) in the group treated with GnRH agonist alone, but did not change in the group treated with GnRH agonist plus MPA. Once again, the effectiveness of GnRH agonist was reversed by a high dose of progestin administration (MPA 20 mg/day).

In 1999, the add-back consensus working group recommended use of appropriate add-back therapy with GnRH agonist treatment to improve the hypoestrogenic symptoms and potentially extend the duration of therapy while preserving therapeutic efficacy [40]. Based on results from RCTs in women with endometriosis, the progestin norethindrone acetate (NETA), known as norethisterone acetate in Europe, was approved by the Food and Drug Administration at a daily dose of 5 mg, combined with synthetic GnRH agonist (leuprolide acetate), as add-back therapy in women with endometriosis [41]. The ESHRE guidelines stated that progestogen only as an add-back therapy does not preserve bone mineral density (BMD) [42].

Chwalisz et al. believed that the inconsistent results obtained in some studies are due to confusion and the multitude of add-back regimens evaluated to date [41]. It should nevertheless be stressed that in vivo, NETA exhibits strong tissue-specific progestogenic, estrogenic or antiestrogenic and androgenic effects and the mean conversion ratio by aromatization of NETA to ethynyl estradiol is 0.7% to 1% at doses of 5 mg NETA [43]. According to Chwalisz et al., the estrogenic activity of NETA may explain its favorable impact on BMD [41].

However, endometriosis and uterine fibroids are different diseases. Hence, the optimal dose for each indication should be determined. It is recommended to use the minimal dose of progestogens (combined with 1 mg E2) to reach the primary endpoint (decrease in fibroids, HMB or both), while preventing BMD loss.

### 3.3. Clinical Evidence in Postmenopausal Women

Having clearly demonstrated the clinical evidence in women of reproductive age, it is also logical to pursue additional investigations into the action of progestogens in postmenopausal women treated with estrogens and progestogens. Indeed, in postmenopausal women with uterine leiomyomas given 2 mg/day of micronized E2, significant changes in mean uterine leiomyoma size were detected in the group treated with 5 MPA mg daily vs. 2.5 mg, revealing the dose-dependent impact of progestogens on fibroid growth. Based on their studies, Palomba et al. and Sener et al. strongly advocated evaluation of different doses of MPA in order to administer the smallest effective dose of progestin during hormone replacement therapy to minimize the risk of fibroid growth [44,45].

Moro et al. reviewed 17 papers (1122 participants) to assess and ascertain the effects of hormone replacement therapy on leiomyoma development and growth in post-menopausal women [46]. They reported that some combinations of estrogen and progestins resulted in a significant increase in fibroid size in relation to the dose of progestin compounds [45,47,48]. These studies also confirm the pivotal role of progesterone and progestogens in leiomyoma growth.

### 3.4. Indirect Proof: Efficacy of Antiprogesterone (Mifepristone) and Selective Progesterone Receptor Modulators

Mifepristone is an antiprogesterone that acts through the inhibition of PRs. Daily administration of 5 and 10 mg of mifepristone yielded uterine volume reduction of 48% after 6 months and 52% after one year [49]. By modulating the progesterone pathway, SPRMs may exert either an agonistic or antagonistic effect on PRs [30,50,51,52,53,54,55,56,57]. Their binding allows these receptors to interact with coactivators and/or corepressors. This is further impacted by the presence of coregulators in a particular cell type, which will dictate whether an SPRM acts more as an agonist or antagonist [26,50,51].

Ulipristal acetate (UPA) was shown to effectively and significantly reduce menstrual bleeding (as assessed by PBAC scores), induce amenorrhea, and decrease the size of leiomyomas by up to 50% after 6 months via its antagonist action on myomas level [56,57,58]. Courtoy et al. described the specific impact of SPRMs (UPA) on myomas. Increased apoptosis, reduced survival and lower proliferation rates were also evidenced by gene expression changes, as was an increase in ECM resorption due to the high activity of matrix metalloproteinases [59,60,61].

UPA was actually a very effective drug [56,57,58] but, unfortunately, due to very rare (1/150,000) but non-predictable cases of drug-induced liver injury (DILI), the CHMP very recently decided to significantly limit its use to premenopausal women who cannot undergo surgery or in the case of uterine fibroid embolization, or if the surgical procedure fails (still pending EMA confirmation).

## 4. Evidence from Available and Recent Systematic Reviews 

In 2013, Sangkomkamhong conducted a systematic review (Cochrane library) on progestogen use in fibroid therapy [33]. Progestins have been utilized for many years in the treatment of uterine fibroids and are still used in some countries (Table 1). However, as emphasized in this Cochrane review, the lack of high-quality studies has proved to be a common problem when systematic evaluation of their benefits and potential harms is required. The authors concluded that evidence is insufficient to support the use of progestogens in treating premenopausal women with uterine fibroids. The same conclusion was reached by Lethaby et al. in their systematic review published in 2017 in the Cochrane library [62].

An extensive review by Bitzer et al. on the medical management of HMB found the LNG-IUS (levonorgestrel-releasing intrauterine system) to be the first-line medical therapy for HMB due to dysfunctional uterine bleeding (characterized by the absence of fibroids). In the presence of HMB due to fibroids, however, it shows much more limited efficacy. These authors reported that MPA and NETA are approved in many countries for the treatment of various forms of “abnormal” uterine bleeding, but their long-term use in fibroid-related HMB is not currently supported by solid evidence, because of the absence of benefits reported in the literature [63].

In a systematic review and network meta-analysis of RCTs investigating medical therapy for uterine fibroids, Gurusamy et al. identified 75 RCTs among 4237 references [64]. Only one reported the results of a progestogen, namely the study by Verspijck et al. previously discussed in this manuscript. Since 2000, there have been no reports of RCTs on progestogens in medical therapy for uterine fibroids [65].

After appraising all available options, Sohn et al. concluded from a literature review and consensus of expert opinion that GnRH agonist and SPRMs are currently the best effective medical therapies, with the best evidence to support their ability to reduce fibroid volume and HMB. Nevertheless, there is a lack of data on the true efficacy of progestogens, which may even promote uterine fibroid growth [66].

## 5. Conclusions

In this review of the most significant papers on the relationship between uterine fibroids and progesterone/progestogens (Table 1), we have clearly shown biochemical, histological, and clinical evidence that progesterone and progestogens play a critical role in the pathogenesis of myomas. In their manuscript entitled “Practice guidelines on the management of uterine fibroids”, Vilos et al. did not ever include progestogens in their algorithm, as they felt that evidence of their efficacy was still lacking [81]. Therefore, summarizing studies on progestogens and uterine fibroids overall, we can conclude that the evidence actually points to a lack of evidence of their efficacy.

On the other hand, effective medications such as GnRH agonist and antagonist induce hypoestrogenic symptoms (including progressive BMD loss and vasomotor symptoms), but hormone add-back therapy may well enhance compliance and extend the duration of therapy. The choice of hormone add-back treatment for myomas should aim to exploit the minimal effective dose of progestogens to preserve the therapeutic effects of GnRH agonist and antagonist. Low doses of E2 (1 mg) combined with NETA (0.5 mg) have proved capable of preventing bone loss in early post-menopausal women [82] and those with endometriosis undergoing GnRH agonist therapy [83]. This combination may therefore be considered an option for women with uterine fibroids subjected to long-term GnRH agonist or antagonist therapy.

## Figures and Tables

**Table 1:** Describe the most relevant studies on the topic.

**A: Progesterone—Progestogens**		
**Authors and References**	**Title**	**Conclusion **
Andersen et al. Baillieres Clin Obstet Gynaecol. 1998 Jun; 12 (2): 225–43. [1]	Factors in fibroid growth	Progesterone increases the mitotic rate of the tumors in vitro and may induce the production of growth factors and/or their respective receptors during the luteal phase.
Rozenbaum, H Contracept Fertil Sex 1989; 17: 153–6. [33]	Traitement médical des fibromes utérins par un progestatif de synthèse du groupe norprégnane	Nomegestrol acetate does not induce significant changes on myoma volume.
Audebert et al. Gynécologie 1989; 40: 23–6. [34]	Utilisation de la promegestone dans le traitement des fibromyomes compliqués de ménométrorragies. Bilan d’une étude multicentrique.	Some patients experienced a decrease in bleeding but no significant change of myoma volume.
Boyd and McCluggage J Clin Pathol. 2011 Jun; 64 (6): 485–9. doi: 10.1136/jcp.2011.089664. Epub 2011 Mar 11. [35]	Unusual morphological features of uterine leiomyomas treated with progestogens	Some morphological factors are described following progestogens therapy. Pathologists should be aware of these progestogen-associated features when reporting uterine leiomyomas whether or not the clinician has indicated that the woman is taking progestogens since otherwise a diagnosis of leiomyosarcoma or smooth muscle tumor of uncertain malignant potential may be rendered.
Amadio E. Abstract Gynecol 1991; 69: 1–4. [67]	Traitement médical des fibromyomes utérins par le nomégestrol acétate.	Among 18 women treated by cyclic progestogens, 10 stopped the treatment because of persistent symptoms
Johnson et al. Int J Gynaecol Obstet 2004 May; 85 (2): 174–6. doi: 10.1016/j.ijgo.2003.09.010. [68]	Depo medroxyprogesterone acetate (DMPA) therapy for uterine myomata prior to surgery	In the group of women treated by DMPA for 3 months before surgery, no improvement was found and some women experienced more severe menorrhagia.
Lumbiganon et al. Br J Obstet Gynaecol. September 1996; 103 (9): 909–14. [69]	Protective effect of depot-medroxyprogesterone acetate on surgically treated uterine leiomyomas: a multicentre case--control study	Prevention of DMPA against uterine leiomyomas formation was found in a control population.
Marsh et al. Obstet Gynecol Clin North Am. 2006 Mar; 33 (1):59–67. doi: 10.1016/j.ogc.2005.12.001. [70]	Steroid hormones and leiomyomas	The sex steroid hormones estrogens and progesterone play an important role in myoma maintenance and growth as evidenced by clinical, molecular, biological, and pharmacological models.
Maruo et al. Hum Reprod Update May–Jun 2004; 10 (3):207–20. doi: 10.1093/humupd/dmh019. [71]	Sex steroidal regulation of uterine leiomyoma growth and apoptosis	This review demonstrates that leiomyoma growth is integrally regulated by the complex cross-talk between sex steroid hormones and growth factors.
Maruo et al. Steroids Oct-Nov 2000; 65 (10–11): 585–92. doi: 10.1016/s0039-128x(00)00171-9. [72]	Effects of progesterone on uterine leiomyoma growth and apoptosis	Bcl-2 protein expression in leiomyoma cells was up-regulated by P4, but down-regulated by E2. Therefore, it seems likely that P4 may also participate in leiomyoma growth through the induction of Bcl-2 protein in leiomyoma cells.
Rein et al. Am J Obstet Gynecol. 1995 Jan; 172 (1 Pt 1):14–8. doi: 10.1016/0002-9378(95)90077-2. [73]	Progesterone: a critical role in the pathogenesis of uterine myomas	Several clinical trials demonstrate that progestins inhibit and/or reverse the ability of hypoestrogenism to shrink uterine myomas, suggesting a critical role for progesterone in growth of myomas. A new hypothesis to explain the pathogenesis of myomas is presented.
Venkatachalam et al. Journal of Obstetrics and Gynaecology (October 2004) Vol. 24, No. 7, 798–800. [74]	Medical management of uterine fibroids with medroxyprogesterone acetate (Depo Provera): a pilot study	In a pilot study, 20 women were treated by depot proverara (DMPA). Although some have an improved bleeding, their study was stopped because of side effects.
**B: Progestogens vs. GnRH Agonist**		
**Authors and References**	**Title**	**Conclusion**
E Verspyck et al. Eur J Obstet Gynecol Reprod Biol. 2000 Mar; 89(1): 7–13. [65]	Leuprorelin depot 3.75 mg versus lynestrenol in the preoperative treatment of symptomatic uterine myomas: a multicentre randomised trial	This study is the only RCT comparing progestogens with GnRHa. Leuprorelin was more effective than lynestrenol because of its more intense antigonadotrophic activity. This RCT was judged as very poor quality in Cochrane review (Sangkomkamhang et al.). Moreover, 22.7% were lost of FU in the lynestrenol group.
**C: GnRH Combined with Progestogens**		
**Authors and References**	**Title**	**Conclusion**
Friedman et al. The Journal of Clinical Endocrinology and Metabolism, Volume 76, Issue 6, 1 June 1993, Pages 1439–1445, https://doi.org/10.1210/jcem.76.6.8501148. [37]	A prospective, randomized trial of gonadotropin-releasing hormone agonist plus estrogen-progestin or progestin “add-back” regimens for women with leiomyomata uteri	After 12 weeks of GnRH therapy, the patients were treated by GnRH a and either OP or P (norethindrone 10 mg /d) A significant regrowth was observed by the coadministration of GnRHa and progestins.
Friedman et al. Fertil Steril. 1988 Mar; 49 (3): 404–9. doi: 10.1016/s0015-0282(16)59763-5. [36]	A randomized, double-blind trial of a gonadotropin releasing-hormone agonist (leuprolide) with or without medroxyprogesterone acetate in the treatment of leiomyomata uteri	No significant changes in myoma volume during cotreatment with GnRHa plus MPA.
Carr et al. J Clin Endocrinol Metab. 1993 May; 76 (5): 1217–23. doi: 10.1210/jcem.76.5.8496313. [38]	An evaluation of the effect of gonadotropin-releasing hormone analogs and medroxyprogesterone acetate on uterine leiomyomata volume by magnetic resonance imaging: a prospective, randomized, double blind, placebo-controlled, crossover trial	GnRHa and MPA coadministration show a correlation with a reversal in the GnRH-a-associated decrease in myomatous tissue volume (GnRH agonist plus MPA). A significant reduction was only observed during GnRHa alone.
West et al. Hum Reprod. 1992 Mar; 7 (3): 328–32. doi: 10.1093/oxfordjournals.humrep.a137643. [75]	Potential role for medroxyprogesterone acetate as an adjunct to goserelin (Zoladex) in the medical management of uterine fibroids	A reduction of 39% in mean uterine volume after 3 months of GnRHa alone vs a reduction of 18% after combined GnRHa + MPA 15 mg daily.
**D: Progestogens in Post Menopause**		
**Authors and References**	**Title**	**Conclusion**
Palomba et al. Eur J Obstet Gynecol Reprod Biol. 2002 May 10; 102 (2): 199–201. [44]	Effect of different doses of progestin on uterine leiomyomas in postmenopausal women	In postmenopausal women with uterine leiomyomas, it is necessary to use the minimal efficacious dose of progestin during HRT because of a higher risk to increase the fibroids dimensions when high doses of progestin are used.
Sener et al. Fertil Steril. 1996 Feb; 65 (2): 354–7. doi: 10.1016/s0015-0282(16)58098-4. [45]	The effects of hormone replacement therapy on uterine fibroids in postmenopausal women	Hormone replacement therapy with 50 micrograms transdermal E2 plus 5 mg MPA increases the size of the uterine fibroids.
**E: Progesterone and Antiprogestin**		
**Authors and References**	**Title**	**Conclusion**
Eisinger et al. J Minim Invasive Gynecol. May-Jun 2005; 12 (3): 227–33. doi: 10.1016/j.jmig.2005.01.022. [49]	Twelve-month safety and efficacy of low-dose mifepristone for uterine myomas	Mifepristone 5 mg and 10 mg/daily reduce uterine volume of 48% after 6 months.
Donnez et al. N Engl J Med 2012; 366:421–432 DOI: 10.1056/NEJMoa1103180. [57]	Ulipristal Acetate versus Leuprolide Acetate for Uterine Fibroids	Both the 5-mg and 10-mg daily doses of ulipristal acetate were noninferior to once-monthly leuprolide acetate in controlling uterine bleeding and were significantly less likely to cause hot flashes.
Bulun N Engl J Med. 2013 Oct 3; 369 (14): 1344–55. doi: 10.1056/NEJMra1209993. [76]	Uterine Fibroids	Antiprogestins induce amenorrhea and reduce fibroid size.
Donnez et al. Fertil Steril. 2015 Feb; 103 (2): 519–27.e3. doi: 10.1016/j.fertnstert.2014.10.038. Epub 24 December 2014 [77]	Efficacy and safety of repeated use of ulipristal acetate in uterine fibroids	Repeated 12-week courses of daily oral ulipristal acetate (5 and 10 mg) effectively control bleeding and pain, reduce fibroid volume, and restore QoL in patients with symptomatic fibroids.
**F: Cochrane/Systemic Review/Meta Analysis**		
**Authors and References**	**Title**	**Conclusion**
Sangkomkamhang et al. Cochrane Database of Systematic Reviews 2013, Issue 2. Art. No.: CD008994. [32]	Progestogens or progestogen-releasing intrauterine systems for uterine fibroids (Cochrane review)	There is insufficient evidence to support the use of progestogens or progestogen-releasing intrauterine systems for uterine fibroids which did not shrink under therapy.
Moro et al. Medicina (Kaunas) 2019 Aug 30; 55 (9): 549. doi: 10.3390/medicina55090549. [49]	The Impact of Hormonal Replacement Treatment in Postmenopausal Women with Uterine Fibroids: A State-of-the-Art Review of the Literature (review)	With uterine fibroids, the choice of the most appropriate HRT regimen is crucial to avoid leiomyomas growth and the symptoms possibly related to it. The minimal effective dose of progestin should be employed as high doses of progestin induce fibroids growth.
Lethaby et al. Cochrane Database Syst Rev. 2017 Nov 15; 11 (11): CD000547. [62]	Preoperative medical therapy before surgery for uterine fibroids (Cochrane review)	Use of preoperative medical therapy before surgery for fibroids is to make surgery easier. There is clear evidence that preoperative GnRHa reduces uterine and fibroid volume.
Bitzer et al. Obstet Gynecol Surv. 2015; 70: 115–30. [63]	Medical management of heavy menstrual bleeding: a comprehensive review of the literature (review)	The LNG-IUS (levonorgestrel-releasing intrauterine system) is the first-line medical therapy for HMB due to dysfunctional uterine bleeding (characterized by the absence of fibroid) but has a much more limited efficacy in presence of HMB due to fibroids.
Gurusamy et al. PLoS ONE 2016 Feb 26; 11 (2): e0149631. doi: 10.1371/journal.pone.0149631. eCollection 2016. [64]	Medical Therapies for Uterine Fibroids—A Systematic Review and Network Meta-Analysis of Randomised Controlled Trials (Network Meta-analysis)	Among the 75 RCT, only one with progestogen was found. Lynestrenol was inferior to GnRHa to treat symptomatic myoma (Verspyck et al.) This study was reported to be of very low quality.
Sohn et al. Obstet Gynecol Sci. 2018 Mar; 61 (2): 192–201. doi: 10.5468/ogs.2018.61.2.192. Epub 13 February 2018 [66]	Current medical treatment of uterine fibroids (review)	There is a lack of high quality evidence assessing the efficacy of progestogens which may even promote uterine fibroid growth.
Koskas et al. J Gynecol Obstet Biol Reprod (Paris) 2011 Dec; 40 (8): 858–74. doi: 10.1016/j.jgyn.2011.09.022. Epub 8 November 2011. [78]	Role of medical treatment for symptomatic leiomyoma management in premenopausal women (review)	Lynestrenol, pregnane and nor-pregnane fail to significantly reduce the fibroid size and the uterine bleeding.
Moroni et al. Annals of Medical and Health Sciences Research | Sep-Oct 2014 | Vol 4 | Special Issue 3 | [79]	Pharmacological Treatment of Uterine Fibroids (Cochrane review)	No evidence supporting the use of Progestogens among 41 studies out of 7658 references. Only 3 studies on the use of progestogens were reported and fail to demonstrate significant benefits.
Moroni et al. Cochrane Systematic Review—Intervention Version published: 20 March 2015 https://doi.org/10.1002/14651858.CD010854.pub2 [80]	Add-back therapy with GnRH analogues for uterine fibroids (review)	In this systematic review on add-back therapy, the mean uterine size in the MPA add back therapy was higher when compared to a GnRH only.
Reis et al. Best Pract Res Clin Obstet Gynaecol. 2016 Jul; 34: 13–24. [30]	Hormones and pathogenesis of uterine fibroids (review)	Progesterone play a major role for uterine fibroids growth and maintenance. Selective progesterone receptors modulators (SPRM’s) have been shown to inhibit fibroid growth.
